# Geriatric Horses in Germany: Approaches to Nutrition, Housing and Overall Care

**DOI:** 10.3390/ani16050813

**Published:** 2026-03-05

**Authors:** Antonia C. Czerner, Arno Lindner, Annette Zeyner, Monika Wensch-Dorendorf, Heidrun Gehlen

**Affiliations:** 1 Equine Clinic, Veterinary Department, Freie Universitaet Berlin, Oertzenweg 19b, 14163 Berlin, Germany; antonia.czerner@fu-berlin.de; 2 Verein zur Förderung der Forschung im Pferdesport e.V., c/o Dr. Arno Lindner, Heinrich-Roettgen-Str. 20, 52428 Juelich, Germany; arnolindner@agpferd.com; 3 Institute of Agricultural and Nutritional Sciences, Martin Luther University Halle-Wittenberg, Theodor-Lieser-Straße 11, 06120 Halle (Saale), Germany; annette.zeyner@landw.uni-halle.de (A.Z.); monika.dorendorf@landw.uni-halle.de (M.W.-D.)

**Keywords:** horse, ageing, survey, feeding, activity, behaviour

## Abstract

Changes in horse management and use mean that a growing number of horses are now kept into older age, while age-specific management practices for these animals remain insufficiently documented. This cross-sectional online survey aimed to describe the housing, feeding, activity, behaviour and care routines of horses aged 20 years and older in Germany, based on 923 questionnaires completed by horse owners about their senior horses. Most horses maintained regular tactile social contact (87.4%, *n* = 805), and over half were still ridden or driven (61.6%, *n* = 566). However, the proportion of horses in active riding or driving use declined strongly with age, from 78% in 20–24-year-old horses to 57% in those aged 25–29 years and 34% in horses aged 30 years or older (*p* < 0.0001). Daily grooming likewise decreased with age, falling from 58% in the youngest age group to 40% in the oldest, while weekly grooming became more common (20–24 vs. ≥30 years: *p* < 0.0001; 25–29 vs. ≥30 years: *p* = 0.0017). Behavioural patterns were largely stable, although the proportion of horses reported to have lost social rank within the herd increased from 25% in 20–24-year-old horses to 41% in horses aged 30 years or older (20–24 vs. 25–29 years: *p* = 0.0050; 20–24 vs. ≥30 years: *p* = 0.0002). Feeding practices also showed pronounced age-related differences: the use of long-fibered dry roughage declined from 90% in the youngest group to 69% in the oldest, whereas the use of pre-chopped dry roughage increased from 21% to 73% (both *p* < 0.0001). The provision of mineral and vitamin supplements likewise decreased with age, from 51% of horses in the youngest group to 35% in the oldest (*p* = 0.0026). These findings indicate consistent differences between age groups in owner-reported management practices and highlight the importance of considering age when evaluating how older horses are managed.

## Simple Summary

In recent decades, more domestic horses appear to be reaching their natural lifespan, leading to a growing population of older animals requiring age-adapted care. However, little is known about how senior horses are managed in daily management practices. To better understand their situation in Germany, a nationwide online questionnaire was conducted among horse owners caring for horses aged over 20 years. The survey asked about housing, feeding, social contact, activity and daily care practices. More than 900 completed responses indicated differences in feeding practices between age groups: older horses were less frequently given long-fibre dry roughage and more frequently given pre-chopped dry roughage, reflecting owner-reported management practices for ageing horses. In addition to these age-related differences in feeding practices, behavioural and social changes were also reported, such as a lower position within the herd. Most older horses were nevertheless kept in social groups, and many remained active, although they were less often used for riding as age increased. Taken together, the data provide an overview of how older horses in Germany are currently kept and managed, and may help owners, veterinarians and trainers to adapt care and management practices to support the health and welfare of ageing horses.

## 1. Introduction

In recent decades, it appears that more domestic horses are reaching their natural lifespan. However, there are no robust, population-level longitudinal studies demonstrating a true rise in biological lifespan in Germany or globally. Instead, the literature consistently documents an increase in the proportion of older horses within equine populations: One contributing factor that must be considered is the increased proportion of horses aged over 16 years participating in German equestrian sport [1]. Comparable trends have also been documented in the United Kingdom in cross-sectional studies investigating geriatric horses aged 15 years and older [2,3].

Similar findings have been described in Australia within surveys of horses aged 15 years and above in Queensland, both with respect to management practices and clinical health status [4,5].

In the United States, studies focusing on horses aged 20 years and older likewise revealed a notable and clinically relevant population of geriatric horses [6,7]. This increase in the proportion of older horses likely reflects improvements in management and veterinary care, rather than a genuine increase in intrinsic lifespan.

The fact that an increasing number of research groups from different parts of the world have independently addressed this topic underscores the growing scientific and practical relevance of geriatric horses in contemporary equine medicine and equestrian sport.

National monitoring data from the United States Department of Agriculture’s National Animal Health Monitoring System indicate that the percentage of the equine population aged 20 years or older increased across study years (1998–2015), further substantiating this trend [8].

Ageing is accompanied by physiological and behavioural changes that influence how these horses should be managed. Population-level data on owner-reported management, feeding strategies, care routines and activity patterns of ageing horses in Germany remain limited.

Maintaining appropriate management becomes even more important as many senior horses (≥15 years of age) remain physically active rather than retiring from work, with a notable proportion still used for training or competition [9].

Veterinarians’ approaches to older horse care are strongly influenced by the owners, who are often the primary observers and decision-makers regarding their animals’ health and welfare [10]. Older horses rely on proactive owners to identify emerging issues, adhere to veterinary advice, and implement age-appropriate interventions, highlighting the central role of education in supporting optimal management [10].

Surveys in Great Britain have shown that most horses are cared for directly by their owners, who manage feeding, turnout and exercise on a daily basis, emphasising their influence on overall welfare [11]. To the authors’ knowledge, comparable population-level data for horses in Germany are lacking, but it is reasonable to assume that the owner-horse relationship is similar. Therefore, understanding management practices of geriatric horses in Germany could provide context-specific insights to guide owner education and promote effective, welfare-oriented care for older horses.

As horses age, they undergo physiological and metabolic changes that can affect digestion and nutrient utilisation [12,13]. These changes often necessitate adjustments in feeding strategies and daily management routines to ensure that geriatric horses maintain health and body condition. Understanding current feeding and management practices is therefore essential for identifying needs, supporting owners and ensuring that care aligns with the requirements of older horses.

To gain insight into the population of older horses in Germany, a questionnaire was developed prior to the 40th Tagung zur Pferdegesundheit (Conference on Equine Health) in 2023—Das alte Pferd im Fokus (“The ageing horse in focus”)—organised by the Verein zur Förderung der Forschung im Pferdesport (Association for the Promotion of Research in Equestrian Sports, FFP). The questionnaire aimed to collect comprehensive information on housing, feeding practices, behaviour, activity and care of horses aged 20 years and older in Germany. By describing how senior horses are currently managed, this study provides data that can inform discussion on management practices and contribute to considerations of welfare-oriented care for the ageing equine population.

Differences in age and horse type (e.g., Warmbloods, ponies, Thoroughbreds) were examined in this study to explore potential variations in owner-reported management practices. It is emphasised that feeding, housing, activity, social contact and behavioural management are primarily shaped by human decisions rather than by the horses’ age or type themselves.

## 2. Materials and Methods

### 2.1. Data Collection

A structured questionnaire was developed to investigate feeding management, housing conditions, social interactions, activity levels and daily care of horses aged 20 years and older (Supplementary Materials). Demographic information, including age, breed, sex and the number of horses over 20 years owned by each respondent, was collected. Diseases, vaccination and deworming routines, as well as dental and hoof care, were documented as part of a separate study.

The survey was created using easyfeedback (easyfeedback GmbH, Koblenz, Germany) and published on the FFP website on 5 April 2023. For the present analysis, only questionnaires referring to a single horse were included (*n* = 923). Questionnaires in which respondents reported on multiple horses were excluded, because individual answers could not be reliably assigned to a specific horse or age category, which would have compromised comparability across cases.

To allow age-related comparisons, horses were grouped into three age categories: 20–24 years (Age group one), 25–29 years (Age group two) and 30 years or older (Age group three). Five-year age intervals were chosen to create groups of approximately comparable size while still capturing meaningful differences between stages of advanced age.

To facilitate the analysis of potential type-related differences in management, owner-reported breeds were assigned to four horse type categories based on commonly used functional and metabolic groupings. These comprised warmbloods, Thoroughbreds/Partbreds, ponies/small horse breeds (robust types). Horses that could not be clearly assigned to any of these three categories were grouped as “others”.

For most questions, no explicit “no answer” or “not applicable” option was provided, so respondents were generally required to choose one of the predefined response options even if they were uncertain or did not wish to disclose information. When individual questions were left unanswered within otherwise completed questionnaires, the respective horse was excluded from the analysis for that variable only but retained for all other analyses. This approach explains the slightly varying absolute numbers across different variables.

Open-ended (free-text) responses were reviewed in full and subsequently grouped into a set of thematically meaningful categories, to which the individual responses were assigned for further analysis.

Social contact was classified according to an adaptation of the AWIN welfare assessment protocol [14]. The AWIN protocol served as a reference framework for categorising social behaviour in this survey-based study.

### 2.2. Statistical Analysis

After completion of the survey, the data were exported from easyfeedback (easyfeedback GmbH) into Microsoft Excel Version 16.89.1 (24091630) (Microsoft Corporation, Redmond, WA, USA) for processing and structuring. Raw entries were carefully cleaned, checked for completeness and systematically organised to ensure a consistent dataset suitable for analysis. Open-text responses were reviewed and assigned to thematic categories, allowing them to be included in the descriptive analyses.

Statistical analyses were performed using SAS Studio (SAS Institute Inc., Cary, NC, USA). Descriptive statistics were generated, with categorical variables reported as counts and percentages and continuous variables as mean ± standard deviation (SD). Percentages were calculated based on the number of horses with valid responses to each question.

Comparisons between predefined age groups of horses were conducted using chi-square tests for categorical variables. Differences in proportions between groups were reported, with 95% confidence intervals calculated to quantify the magnitude of these differences. As the study is cross-sectional and exploratory, no formal correction for multiple testing was applied. A significance level of *p* < 0.05 was used.

## 3. Results

### 3.1. Study Population

A total of 923 single-horse questionnaires were initially received. After applying the predefined inclusion criteria, 919 horses aged between 20 and 39 years (mean ± SD: 25.74 ± 3.96 years) were included in the analysis. A total of 42.22% (*n* = 388) were 20–24 years old (Age group one), 38.08% (*n* = 350) were 25–29 years old (Age group two) and 19.70% (*n* = 181) were 30 years or older (Age group three).

The horses included in the analysis were categorised by type as follows: warmbloods accounted for 57.61% (*n* = 530), ponies and small horse breeds for 25.00% (*n* = 230), Thoroughbreds and Partbreds for 9.57% (*n* = 88) and other types for 7.83% (*n* = 72).

With regard to sex, mares accounted for 42.08% (*n* = 388), whereas geldings and stallions represented 57.92% (*n* = 534) of the study population.

### 3.2. Time Commitment and Grooming

A percentage of 18.74% (*n* = 155) spent up to seven hours, 36.64% (*n* = 303) 8–14 h, 28.90% (*n* = 239) 15–21 h and 15.72% (*n* = 130) more than 22 h per week on horse care and management.

**Figure 1:**
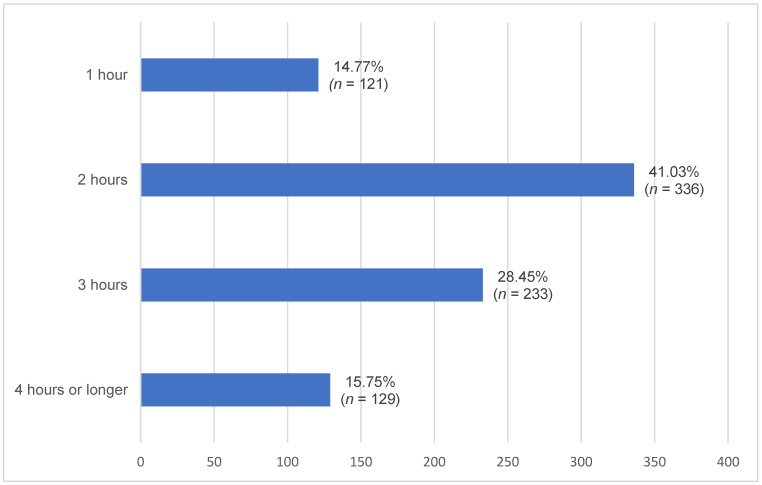
Owner-reported daily time commitment among age groups of geriatric horses (≥20 years). Horizontal bars indicate the number (*n*) and percentage (%) of horses.

**Figure 2:**
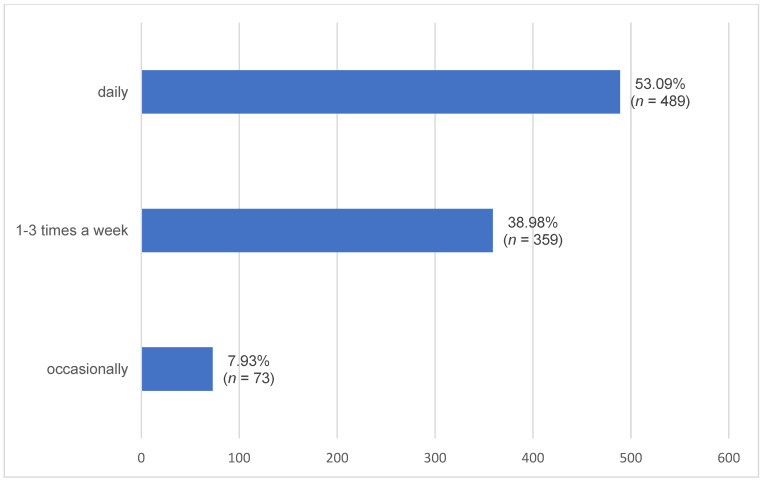
Weekly grooming time reported by owners of geriatric horses (≥20 years). Horizontal bars indicate the number (*n*) and percentage (%) of horses.

Daily time commitment and weekly grooming time are shown in Figure 1 and Figure 2. There were significant differences in owner-reported grooming frequency between age groups one and three, as well as between age groups two and three (Table 1).

In addition, warmbloods (0.57, 95% CI: 0.524–0.608) were groomed significantly more frequently on a daily basis than ponies and small horse breeds (0.48, 95% CI: 0.418–0.547; *p* = 0.0342). Ponies and small horse breeds (0.45, 95% CI: 0.387–0.516), in turn, were groomed significantly more frequently one to three times per week than warmbloods (0.35, 95% CI: 0.310–0.392; *p* = 0.0088).

### 3.3. Housing Systems

**Figure 3:**
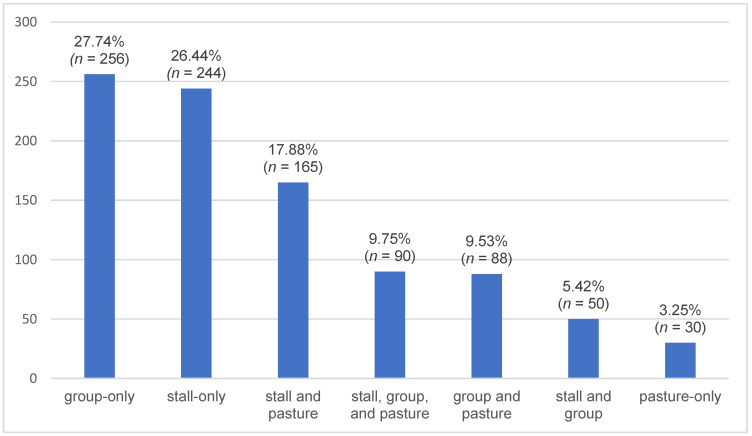
Owner-reported distribution of housing systems for horses aged ≥ 20 years, showing proportions of stall, group and pasture housing (percentages with sample size n). Horses could be included in more than one category.

Three housing categories were distinguished: stall housing (indoor stalls, outdoor stalls, paddock stalls or exercise stalls), group housing (comprising open stalls, run-in shelters, trails, active stalls and paddocks) and pasture housing. The distribution of the owner-reported housing systems is shown in Figure 3.

Considering the three categories individually (with overlaps), 59.48% (*n* = 549) of horses experienced at least temporary stall housing, 52.44% (*n* = 484) were kept in some form of group housing and 40.41% (*n* = 373) had access to pasture. There was no significant change in the choice of housing system with age.

There was a significant difference in stall (*p* < 0.0001) and group (*p* < 0.0001) housing between horse types: ponies and small horse breeds (0.64, 95% CI: 0.579–0.703) were more frequently kept in group housing than warmbloods (0.47, 95% CI: 0.424–0.509) as well as more frequently than Thoroughbreds and Partbreds (0.51, 95% CI: 0.408–0.614).

In contrast, warmbloods (0.66, 95% CI: 0.617–0.698) as well as Thoroughbreds and Partbreds (0.63, 95% CI: 0.520–0.720) were more frequently kept in stall housing than ponies and small horse breeds (0.45, 95% CI: 0.389–0.517).

### 3.4. Social Contact

Most horses (87.40%, *n* = 805) had owner-reported social contact through tactile interactions within a group. 10.53% (*n* = 97) had tactile contact through a fence and 2.06% (*n* = 19) experienced only visual, auditory and olfactory contact.

Horses in age group 1 (0.90, 95% CI: 0.869–0.929) were significantly more likely to have social contact through tactile interactions within a group than those in age group 2 (0.85, 95% CI: 0.851–0.899; *p* = 0.0390).

No significant association was found between horse type and the type of social contact.

### 3.5. Activity

The reported distribution of retired and non-retired horses is shown in Table 2. Horses still in work were reported by their owners to perform activities such as leisure riding, trail riding, jumping, dressage, eventing, endurance riding, vaulting, driving, and Western riding. The proportion of horses reported to be used for riding varied with age (Table 3). Horse type was not associated with differences in owner-reported riding activity.

### 3.6. Behavioural Parameters

Owners reported patterns in five different behavioural parameters (Table 4). The position/rank within group/herd showed a significant association with age (Table 5). No significant association was found between horse type and behavioural parameters.

### 3.7. Feeding

The frequency of feedings per day varied among respondents (Table 6).

Regarding the type of feed, 86.25% (*n* = 740) of horse owners provided roughage in combination with concentrate feed and 13.75% (*n* = 118) provided roughage without concentrate feed. Type of feed provided by the owner differed significantly between horse types (<0.0001):

Ponies and small horse breeds (0.75, 95% CI: 0.692–0.806) were less frequently fed concentrate feed in addition to roughage than both warmbloods (0.90, 95% CI: 0.869–0.923) and Thoroughbreds and Partbreds (0.93, 95% CI: 0.848–0.967).

**Figure 4:**
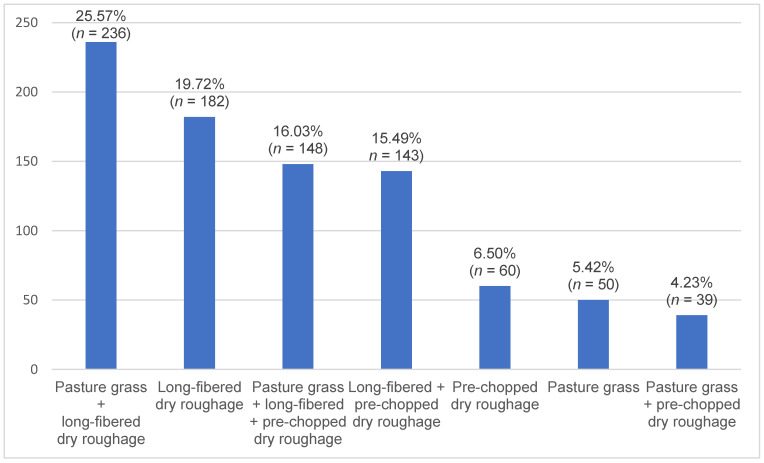
Owner-reported distribution of long-fibered dry roughage, pre-chopped dry roughage, pasture grass and their combinations in the study population.

The use of different types of roughage was as follows: 55.13% (*n* = 473) of respondents used pasture grass, 82.63% (*n* = 709) used long-fibered dry roughage and 45.45% (*n* = 390) used pre-chopped dry roughage (soaked if necessary). Some respondents used combinations of feed types, resulting in overlaps. Roughage feeding practices and their combinations are shown in Figure 4. The feeding of long-fibered and pre-chopped dry roughage was significantly age-dependent (Table 7).

Regarding the provision of mineralised and vitamin-enriched complementary feed, 44.0% (*n* = 406) of respondents reported providing such feed to their horses.

Supplementation with this feed decreased significantly with age, differing between age groups (*p* = 0.0026): Age group one: 0.51, 95% CI: 0.455–0.555; age group two: 0.43, 95% CI: 0.381–0.485; age group three: 0.35, 95% CI: 0.285–0.425.

Horses that were still ridden (0.48, 95% CI: 0.439–0.522) were significantly more likely to receive mineralized and vitamin-enriched complementary feed than retired horses (0.39, 95% CI: 0.340–0.443; *p* = 0.0089).

## 4. Discussion

This study provides a comprehensive overview of housing, feeding, care routines, activity levels and behavioural characteristics in horses aged 20 years and older. By analysing survey data from a large population of geriatric horses, clear age- and type-related trends were identified across multiple management domains. These findings contribute to a better understanding of how owners adapt their practices to the changing needs of older horses and highlight areas where welfare-oriented management can be supported.

### 4.1. Time Commitment and Grooming

Most horses in our study received between two and three hours of daily care, with over half of the horses groomed daily. Younger horses (age groups one and two) were reported to be groomed more frequently than older horses (age group three). This difference likely reflects owner management practices, such as allocating more time and attention to younger animals or perceiving their care as requiring greater effort, rather than intrinsic differences in the horses’ activity levels.

No clear explanation could be identified for the lower grooming frequency reported for ponies and small horse breeds compared with warmbloods. Experimental studies have shown that tactile or vocal contact from humans during short-term social isolation can influence behavioural and physiological responses in horses, with differences between sexes and types of contact [15]. These findings provide contextual information but cannot be directly applied to the present dataset, as human–horse interactions were not assessed using standardised or validated methods.

### 4.2. Housing Systems

Regarding housing, stall housing was reported for 59.48% of horses at least temporarily, while 52.44% were kept in group housing and 40.41% had access to pasture. No clear trend in the housing system was observed with increasing age in our dataset, as horses were reported to be kept in overlapping arrangements of stall, group and pasture housing. While another German study suggested that younger horses are more often kept individually and older horses (≥15 years) more frequently in groups [16], our findings indicate that such distinctions may not be strictly applicable, given the variability and overlap in the owner-reported housing practices.

An Austrian study found that horses kept in open group paddocks displayed a more even daily distribution of feeding and movement than horses kept in more restrictive systems [17]. In addition, a recent systematic review and meta-analysis of equine time-activity budgets demonstrated that feeding, resting and locomotion differ across management systems, social housing and feeding regimes, with grouped, free-ranging and grazing horses spending more time feeding and moving than stabled or isolated horses [18]. These external findings provide a broader context for interpreting how management may influence daily activity patterns, but they cannot be directly linked to the horses in the present survey.

Within our study population, the largest proportion of horses (27.74%) was reported to be kept in full-day group-only housing. However, 59.48% of respondents indicated that their horse was kept individually for at least part of the day. These overlapping categories were handled descriptively in the analysis, reflecting owner-reported practices without assuming exclusive housing arrangements or inferring causal relationships. Ponies and small horse breeds were more often kept in group housing, which corresponds to recommendations for robust breeds such as Shetland ponies under appropriate management conditions [19].

### 4.3. Social Contact

With respect to social contact, 12.59% of horses were reported to have limited tactile contact, whereas 87.40% had tactile contact within a group, based on an established classification system [14,20]. These figures describe the distribution of owner-reported contact opportunities. Experimental studies have shown that horses restricted to visual contact display more abnormal behaviours than those with full social interaction [21], but behavioural outcomes of this type were not assessed here. Horses aged 20–24 years were reported to have more tactile social contact than those aged 25–29 years. Increasing age and repeated exposure to tactile stimuli have been discussed in the literature in relation to habituation and reduced reactivity [22], but in the present study, social contact arrangements were determined by owners and management conditions rather than by the horses themselves.

Owner-reported activity differed across age groups, with fewer horses reported to be ridden or driven at advanced age, particularly among horses aged 30 years and older. Training studies have demonstrated that although older horses show slower cardiovascular recovery and reduced autonomic responses compared to younger horses, structured exercise can partially reverse age-related declines in aerobic capacity [23,24]. These findings suggest that decreased activity in older horses in this study may reflect management decisions rather than absolute physiological limitations.

Most behavioural traits were reported to remain stable across age groups, whereas changes in social rank were more often reported in older horses. These data reflect owner perceptions of herd position and do not represent validated measures of dominance, social competence or welfare. Factors such as pain, chronic disease, group composition and management decisions may influence social dynamics, but these were not evaluated here. Recommendations for the management of older horses therefore remain based on general housing principles, such as the provision of adequate and protected lying areas [25]. Large behavioural datasets have described age-related differences in personality traits, with older horses reported as bolder or more independent [26], but these findings originate from different populations and study designs. Overall, the present results provide a descriptive overview of owner-reported care, housing, social contact and activity without allowing inferences about emotional state, welfare or social functioning.

### 4.4. Feeding

In the present survey, owner-reported feeding practices differed across age groups. With increasing age, horses were less often fed long-fibered dry roughage, whereas pre-chopped dry roughage was more commonly reported for older animals. In addition, the use of mineralised and vitamin-enriched complementary feeds was reported less often in older and retired horses, and ponies/small horse breed were given smaller amounts of concentrate. Although information on dental status and disease was collected in the questionnaire, these variables are analysed in a separate publication and were therefore not included in the present evaluation. Consequently, no conclusions can be drawn here regarding the nutritional adequacy of the reported feeding practices.

The shift towards modified roughage forms in older horses may reflect practical feeding adaptations to age-related changes such as altered chewing ability or feed intake. However, this interpretation remains tentative because no animal-level clinical data were considered in this analysis. Beyond chewing ability, the physical presentation of forage may also influence posture, feeding behaviour and musculoskeletal loading, particularly in older horses with reduced joint mobility or chronic discomfort. Experimental work has shown that some slow-feeding devices, such as hay nets, can promote sustained cervical flexion and increase frustration-related behaviours compared with feeding systems that allow a more elongated neck posture [27]. Such findings provide a behavioural and biomechanical context for the use of chopped forage and alternative feeding devices, but their relevance for the present population cannot be assessed from the available data.

Similarly, the lower reported use of complementary feeds in older and retired horses may reflect simplified feeding regimes or owner perceptions of reduced supplementation needs, but this cannot be verified within the scope of the present data.

In the literature, age-related changes in dentition and digestive physiology have been described that may make the processing of coarse or fibrous feed more difficult for older horses [28]. Experimental studies have further shown that older horses, when fed identical diets, can display lower apparent digestibility of crude protein and fibre and reduced phosphorus retention compared with younger horses, while calcium digestion remains largely unaffected [12]. In addition, forage physical quality has been identified as a relevant factor, as overly mature hay and sharp plant structures can cause oral lesions and stomatitis, potentially exacerbating dysphagia in horses with advanced dental wear [29]. These findings suggest that owners’ choices of chopped or softened roughage may relate not only to chewing efficiency but also to the avoidance of mechanical irritation of the oral cavity, although this cannot be examined within the present dataset. These findings provide a physiological context for the feeding practices reported here, but they cannot be directly linked to the horses included in this survey.

Current feeding guidelines state that most older horses do not require special diets unless body condition declines, whereas failing aged horses may benefit from rations with higher protein content, adjusted Ca:P ratios, reduced fibre and high digestibility, as well as moistened or pelleted feeds to compensate for poor dentition [13,30]. These recommendations are cited here to place the reported practices within an established framework, but the present study does not allow an assessment of whether the feeding regimes met these criteria. Given the frequent use of concentrates to support body condition in senior horses, experimental evidence indicating that high-starch diets alter gastrointestinal microbial populations and may increase digestive instability compared with high-fibre diets provides additional context for interpreting such management strategies [31], without allowing conclusions for the present population.

It should also be considered that group-housing systems, which are commonly used for older horses, may limit the feasibility of individualised feeding adjustments [32], and that age-related diseases could further complicate nutritional management [33]. While information on health status and dentition was available in the overall dataset, it was not included in this analysis and could therefore not be taken into account here.

Ponies have lower metabolizable energy maintenance requirements and utilise dietary energy more efficiently than horses [34], which may provide a physiological context for the lower amounts of concentrate reported for ponies in this study. Recent experimental work has also shown that ponies differ from horses in mouth morphology and chewing strategies, which can influence intake rate and interaction with feeding devices [35], offering additional context for the observed management differences. Experimental data further show that cereal- and fat-rich diets can increase total intake and nutrient digestibility across breeds [36], but these findings are independent of the owner-reported feeding practices described here.

The reported use of mineralised and vitamin-enriched complementary feeds was lower in older and retired horses, a pattern that is similar to findings from an Australian study [4]. This, however, does not fully align with current feeding recommendations for older horses, which suggest that rations should primarily consist of high-quality roughage complemented by appropriate mineral and vitamin supplements [37].

### 4.5. Limitations

This study was based on self-reported data provided by horse owners without veterinary verification, which may involve some degree of recall bias or misclassification. However, this approach allowed the inclusion of a large and diverse group of respondents. Participation was voluntary and likely attracted particularly engaged and motivated owners, which may introduce some selection bias and should be considered when interpreting the generalisability of the findings.

The survey did not collect detailed quantitative feeding data, and no clinical, welfare-related, or performance outcome measures were included. The results therefore primarily reflect reported management practices rather than objectively measured health or welfare outcomes.

The cross-sectional design captures management practices at a single point in time and does not permit causal inference or the assessment of temporal changes. In addition, environmental and seasonal factors were not controlled for, and some housing characteristics, such as box size or pasture quality, were not available.

Finally, open-ended responses were categorised for analysis, which facilitated systematic evaluation but may have reduced some of the original nuance in how management practices were described.

## 5. Conclusions

Overall, the findings provide descriptive insights into owner-reported management and feeding practices of geriatric horses in Germany, including aspects of feeding, housing, activity, social contact and daily care. As this study is cross-sectional and based on self-reported data, it does not directly assess physiological, behavioural or welfare outcomes. These results highlight patterns in reported management practices and may inform future research or discussions on care strategies for older horses, without implying causal relationships or direct effects on horse welfare.

## Figures and Tables

**Table 1:** Descriptive differences in owner-reported grooming frequency among three age groups of geriatric horses (≥20 years).

Grooming Frequency	Age Group (Years)	Owner-Reported Proportion	95% CI	*p*-Value
Daily	20–24 vs. 25–29	0.58	0.530–0.628	0.3090
		0.54	0.489–0.594	
	20–24 vs. ≥30	0.58	0.530–0.628	<0.0001
		0.40	0.327–0.469	
	25–29 vs. ≥30	0.54	0.489–0.594	0.0017
		0.40	0.327–0.469	
1–3 times a week	20–24 vs. 25–29	0.34	0.296–0.391	0.4627
		0.37	0.319–0.420	
	20–24 vs. ≥30	0.34	0.296–0.391	<0.0001
		0.54	0.463–0.609	
	25–29 vs. ≥30	0.37	0.319–0.420	0.0002
		0.54	0.463–0.609	
occasionally	20–24 vs. 25–29	0.08	0.051–0.104	0.5785
		0.09	0.059–0.119	
	20–24 vs. ≥30	0.08	0.051–0.104	0.6339
		0.07	0.030–0.103	
	25–29 vs. ≥30	0.09	0.059–0.119	0.3677
		0.07	0.030–0.103	

**Table 2:** Owner-reported activities of horses aged ≥20 years, showing proportions of retired and non-retired horses, including subcategories for ridden/driven and groundwork (n for each category in parentheses).

Category	Number of Horses	Description
Non-retired horses	61.59% (*n* = 566)	Horses that are still ridden or driven
Proportion ridden/driven and ground-worked	59.52% (*n* = 547)	Horses that are both ridden/driven and exercised on the ground
Proportion only ridden or driven	2.07% (*n* = 19)	Horses exclusively ridden or driven, no groundwork
Retired horses	38.41% (*n* = 353)	Horses no longer used for riding or driving
Proportion completely inactive	5.88% (*n* = 54)	Horses not engaged in any activity
Proportion ground-worked or walked, but not ridden	32.54% (*n* = 299)	Horses still receiving groundwork

**Table 3:** Owner-reported proportion of horses aged ≥20 years used for riding by age group (20–24, 25–29, ≥30 years) with 95% CI.

Age Group (Years)	Owner-Reported Proportion	95% CI	*p*-Value
20–24	0.78	0.738–0.820	<0.0001
25–29	0.57	0.522–0.625	
≥30	0.34	0.273–0.411	

**Table 4:** Owner-reported patterns in behavioural parameters of geriatric horses (≥20 years), showing proportions of horses with decreased, unchanged or increased responses for skittishness, compatibility with other horses, position/rank within group/herd, compatibility with owner, and compatibility with other humans (percentages with n).

Behavioural Parameter	Decreased	Remained the Same	Increased
Skittishness	17.28% (*n* = 145)	65.32% (*n* = 548)	17.40% (*n* = 146)
Compatibility with other horses	10.00% (*n* = 89)	78.76% (*n* = 701)	11.24% (*n* = 100)
Position/Rank within group/herd	31.52% (*n* = 278)	60.43% (*n* = 533)	8.05% (*n* = 71)
Compatibility with the owner	0.77% (*n* = 7)	66.37% (*n* = 602)	32.86% (*n* = 298)
Compatibility with other humans	5.12% (*n* = 46)	82.20% (*n* = 739)	12.68% (*n* = 114)

**Table 5:** Owner-reported changes in position/rank within group/herd by age group (≥20 years), showing proportions of horses with decreased, unchanged, or increased position/rank and 95% confidence intervals.

Position/Rank Within Group/Herd	Age Group (Years)	Owner-Reported Proportion	95% CI	*p*-Value
Decreased	20–24 vs. 25–29	0.25	0.205–0.293	0.0050
		0.35	0.294–0.396	
	20–24 vs. ≥30	0.25	0.205–0.293	0.0002
		0.41	0.333–0.479	
	25–29 vs. ≥30	0.35	0.294–0.396	0.1795
		0.41	0.333–0.479	
Remained the same	20–24 vs. 25–29	0.63	0.583–0.682	0.4320
		0.60	0.551–0.656	
	20–24 vs. ≥30	0.63	0.583–0.682	0.0339
		0.54	0.463–0.611	
	25–29 vs. ≥30	0.60	0.551–0.656	0.1491
		0.54	0.463–0.611	
Increased	20–24 vs. 25–29	0.12	0.086–0.152	0.0014
		0.05	0.027–0.075	
	20–24 vs. ≥30	0.12	0.086–0.152	0.0242
		0.06	0.023–0.092	
	25–29 vs. ≥30	0.05	0.027–0.075	0.7712
		0.06	0.023–0.092	

**Table 6:** Owner-reported number of feedings per day in geriatric horses (≥20 years), showing proportions and sample size (n) for one to five feedings per day.

Feedings per Day	One Time	Two Times	Three Times	Four Times	Five Times
Number of horses	12.96% (*n* = 116)	36.09% (*n* = 323)	34.41% (*n* = 308)	8.16% (*n* = 73)	8.38% (*n* = 75)

**Table 7:** Owner-reported proportions of horses receiving long-fibered or pre-chopped dry roughage across age groups (20–24, 25–29, and ≥30 years), with 95% confidence intervals and *p*-values.

Type of Roughage	Age Group (Years)	Owner-Reported Proportion	95% CI	*p*-Value
Long-fibered dry roughage	20–24	0.90	0.867–0.929	<0.0001
	25–29	0.82	0.771–0.855	
	≥30	0.69	0.615–0.753	
Pre-chopped dry roughage	20–24	0.21	0.173–0.258	<0.0001
	25–29	0.57	0.521–0.628	
	≥30	0.73	0.658–0.791	

## Data Availability

Data are available from the authors upon reasonable request.

## Supplementary Materials

The following supporting information can be downloaded at: https://www.mdpi.com/article/10.3390/ani16050813/s1, File S1: Online questionnaire.
