# Supplementary material for: Geriatric Horses in Germany: Approaches to Nutrition, Housing and Overall Care
Source: Animals (Basel). 2026 Mar 5;16(5):813. doi: 10.3390/ani16050813 (PMC12984791; doi:10.3390/ani16050813)
Supplement: Supplementary file 1 [file animals-16-00813-s001.zip › animals-4120828-supplementary.pdf]

Survey in Preparation for the 40th FFP Conference on Equine Health: Focusing on the Older Horse

Welcome to our survey!
Thank you for taking a few minutes to participate. We look forward to your observations and experiences with older horses.

This survey will run until 15 June 2023 or will be closed earlier if the maximum number of responses is reached.

The results of the survey will be presented at our 40th Annual Conference from 7–9 July 2023 in Espenau near Kassel. We are eager to see your responses.

Let's get started!

How many horses aged 20 years or older do you own?

- Number
- If more than 10, how many?

How old is your oldest horse?

- Age in years

Breed

Sex

What form of husbandry does your horse/do your horses live in? (Multiple answers possible)

- **Stall**
    - ☐ Indoor
    - ☐ Outdoor
    - ☐ Paddock
    - ☐ Active
- **Group (free-stall)**
    - ☐ Enclosed without separate functional areas
    - ☐ Enclosed with separate functional areas
    - ☐ Open without separate functional areas
    - ☐ Open with separate functional areas (Active Stable)
- **Pasture**

    - ☐ with a weather shelter
- **Other**

Does your horse have/do your horses have social contact with other horses?

- Social contact differs as follows:
    - ☐ No social partners (lives alone)
    - ☐ No visual contact with other horses
    - ☐ Visual, auditory, and olfactory contact
- Tactile contact possibilities
    - ☐ Over the fence
    - ☐ Group

Which activities do you carry out with your horse/horses?

**Activity**	**Several times a week**	**Weekly**	**Fortnightly**	**Monthly**	**Irregularly**	**Not specified**
Recreational riding						
Trail riding						
Jumping						
Dressage						
Eventing						
Endurance riding						
Vaulting						
Driving						
Groundwork						
Western riding						
Going for a walk						

How much time do you spend with your horses?

- Indicate in hours per day
- Indicate in hours per week

Which change(s) have taken place in your horse/horses with age?

	Increased	Remained the same	Decreased	Particularly noticeable	Minor	No information
**Mobility**						
**General Medicine** (e.g., infectious diseases)						
**Respiratory diseases**						
**Ophthalmology** (e.g., eye problems, inflammations, injuries, etc.)						
**Dermatology** (e.g., skin diseases, parasite infestation, sweet itch, etc.)						
**Hearing problems**						
**Internal Medicine** (e.g., colic, digestive problems)						
**Circulatory disorders**						
**Neurology** (e.g., movement disorders, behavioural changes, coordination problems, etc.)						
**Orthopaedics** (e.g., joint problems, lameness, hoof problems, etc.)						
**Dentistry** (e.g., dental problems, tartar, temporomandibular joint problems, etc.)						

Have you observed behavioural changes, and if so, which ones?

**Behavioural aspect**	**Increased**	**Remained the same**	**Decreased**	**Particularly noticeable**	**Minor**	**No information**
Startle response/ Frightfulness						
Compatibility with other horses						
Position/Rank in the group/herd						

Compatibility with people other than you						
Compatibility with you personally						

Have you changed anything in the care (feeding, keeping, use, or medical support) over the years?

- ☐ Yes
- ☐ No

Changes in Care

- What have you changed in the care and, if applicable, how?
  - The feeding
  - The keeping/husbandry
  - The use (work/activity)
  - The support/management
  - The health care/medical support

How often are your horses fed?

- Indicate times per day

Which feeds and quantities are used to create the daily ration?

Do you have your horses vaccinated?

- ☐ Yes
- ☐ No

Vaccinations

- **Influenza**
  - ☐ Once annually
  - ☐ Twice annually
  - ☐ No vaccination specifically against influenza
- **Tetanus**

        - ☐ Once annually
        - ☐ Less frequently than once annually
        - ☐ No vaccination against tetanus
    - **Other Vaccinations**
        - Please name your vaccination against:

How often are your horses wormed and according to which worming strategy?

- ☐ Routinely several times annually
- ☐ Once annually
- ☐ Targeted (selective) worming
- ☐ No worming
- ☐ Another schedule, namely:

How often are your horses groomed?

- ☐ Daily
- ☐ 1-3 times a week
- ☐ From time to time

How often are dental checks/treatments carried out?

- ☐ Once annually
- ☐ No checks
- ☐ Another rhythm, namely:

How often is hoof care/trimming carried out?

- ☐ Every 6-8 weeks
- ☐ Every 3 months
- ☐ Less frequently
- ☐ Never

The hoof care/trimming is carried out by:

What applies to your horse/your horses?

- ☐ Horseshoes on four hooves
- ☐ Horseshoes only on the front hooves
- ☐ Barefoot (during turnout and during use)
- ☐ Barefoot (with hoof boots during use)

And finally, our last question:

Do you have special recommendations for other horse owners?
